# Testing the Efficacy of DNA Barcodes for Identifying the Vascular Plants of Canada

**DOI:** 10.1371/journal.pone.0169515

**Published:** 2017-01-10

**Authors:** Thomas W. A. Braukmann, Maria L. Kuzmina, Jesse Sills, Evgeny V. Zakharov, Paul D. N. Hebert

**Affiliations:** Centre for Biodiversity Genomics, Biodiversity Institute of Ontario, University of Guelph, Guelph, Ontario, Canada; Chinese Academy of Medical Sciences and Peking Union Medical College, CHINA

## Abstract

Their relatively slow rates of molecular evolution, as well as frequent exposure to hybridization and introgression, often make it difficult to discriminate species of vascular plants with the standard barcode markers (*rbcL*, *matK*, ITS2). Previous studies have examined these constraints in narrow geographic or taxonomic contexts, but the present investigation expands analysis to consider the performance of these gene regions in discriminating the species in local floras at sites across Canada. To test identification success, we employed a DNA barcode reference library with sequence records for 96% of the 5108 vascular plant species known from Canada, but coverage varied from 94% for *rbcL* to 60% for ITS2 and 39% for *matK*. Using plant lists from 27 national parks and one scientific reserve, we tested the efficacy of DNA barcodes in identifying the plants in simulated species assemblages from six biogeographic regions of Canada using BLAST and mothur. Mean pairwise distance (MPD) and mean nearest taxon distance (MNTD) were strong predictors of barcode performance for different plant families and genera, and both metrics supported ITS2 as possessing the highest genetic diversity. All three genes performed strongly in assigning the taxa present in local floras to the correct genus with values ranging from 91% for *rbcL* to 97% for ITS2 and 98% for *matK*. However, *matK* delivered the highest species discrimination (~81%) followed by ITS2 (~72%) and *rbcL* (~44%). Despite the low number of plant taxa in the Canadian Arctic, DNA barcodes had the least success in discriminating species from this biogeographic region with resolution ranging from 36% with *rbcL* to 69% with *matK*. Species resolution was higher in the other settings, peaking in the Woodland region at 52% for *rbcL* and 87% for *matK*. Our results indicate that DNA barcoding is very effective in identifying Canadian plants to a genus, and that it performs well in discriminating species in regions where floristic diversity is highest.

## Introduction

DNA barcoding employs sequence variation in short, standardized gene regions as a tool to discriminate species [1]. The ideal DNA barcode region is reliably amplified and sequenced across large assemblages of taxa and provides a high level of species discrimination [2]. The success of the 5’ region of the mitochondrial cytochrome *c* oxidase I (COI) gene in discriminating animal species motivated efforts to identify gene regions that might deliver similar resolution for plants. Due to the extremely low rates of nucleotide substitution in mitochondrial genes in most plant lineages [3], COI was not a candidate. However, building on their intense use for phylogenetics and molecular systematics, two plastid gene regions were considered as DNA barcodes for vascular plants and the large subunit of RuBisCo (*rbcL*) in combination with an intron maturase (*matK*) were adopted as standards [4; 5]. Because these regions often fail to resolve congeners [6; 7; 8; 9; 10; 11], there has been a subsequent trend, building on earlier suggestions [12; 13; 14], to couple them with the nuclear-encoded ribosomal internal transcribed spacer, ITS2 [2; 15; 16].

A considerable number of studies have now examined the performance of different markers with respect to both their ease of amplification and their capacity to resolve plant species [9; 10; 15; 17; 18; 19; 7; 20; 21; 22; 23; 24; 25; 26]. This work has indicated that *rbcL* has the highest level of sequence recovery (90–100%), followed by ITS2 (~90%), while *matK* is more difficult (56–90%). The efficacy of these gene regions in discriminating species has been determined by tree-based (phylogenetic) or basic local alignment (BLAST) algorithms. ITS2 has been reported to deliver the highest species resolution (79–93%) followed by *matK* (45–80%), and *rbcL* (17%–92%). It was suggested that the efficacy of DNA barcodes in delivering species-level identifications could be improved by developing local libraries [7; 27], and it was later demonstrated that this approach did indeed improve resolution [9; 23]. The effectiveness of such libraries depends upon complete sampling of local floras, accurate identification of the specimens that are analyzed, and quality of the resultant sequences [28].

Comparisons among past studies are difficult due to high variance in taxonomic scope (30–4800 species), biogeographic focus (e.g. Arctic and temperate floras, tropical trees), the number of DNA barcode markers employed (2–8 chloroplast and nuclear), and the methodologies used for making taxonomic assignments. In fact, no prior study has involved a large-scale comparative analysis of the capacity of the standard barcode markers (*rbcL*, *matK*, ITS2) to deliver a species-level identification for different biogeographic communities using a standard barcode library with the same methods. This study addresses this gap by employing a DNA barcode library for the vascular plants of Canada to determine the method that yields the best species resolution and the marker (*rbcL*, *matK*, ITS2) with the highest performance. As well, this study examines the efficacy of custom DNA barcode libraries for identification success, and compares phylogenetic diversity measures between sites and among species–rich families to determine factors affecting species resolution.

## Materials and Methods

### Taxonomic sampling

Sequences for three DNA barcode regions (*rbcL*, *matK* and ITS2) were generated for the vascular plants of Canada at the Canadian Center for DNA Barcoding [29]. Complete taxonomic information, collection records, voucher images and sequences for 17,995 specimens are publically available through BOLD [30] in the plants of Canada project (Available as of January 4, 2016; doi: dx.doi.org/10.5883/DS-VASCAN). This sequence library includes records for 4923 of the 5108 species of non-hybrid origin (~96%) with coverage for all 1153 genera and 171 families in the Database of Vascular Plants of Canada (VASCAN; [31]). Coverage varies among the three gene regions; the *rbcL* dataset is most complete with 16,008 sequences spanning 4790 species (~93.8%) in 168 families (Table 1). The ITS2 library includes 6630 sequences representing 3044 species (~59.6%) in 125 families while the *matK* dataset includes 6599 sequences covering 2000 species (39%) across 118 families. Overall, 78% of the species (3839) possess records for some combination of two markers, but only 1074 species (22%) have data for all three.

**Table 1:** List of localities, corresponding terrestrial ecozones and biogeographic regions used to test the taxonomic resolution of *rbcL*, *matK*, and ITS2 libraries for the vascular plants of Canada. The number of species at each locale is in parentheses.

Park (species)	Terrestrial Ecozones	Region
Ellesmere Island NP (135)	Northern Arctic	Arctic
Ivvavik NP (402)	Southern Arctic	Arctic
Nahannii NP (652)	Taiga Plains	Arctic
Torngat Mountains NP (368)	Arctic Cordillera	Arctic
Ukkusiksalik NP (150)	Northern Arctic	Arctic
Wapusk NP (272)	Hudson Plains	Arctic
Forillon NP (622)	Atlantic Maritime	Atlantic
Fundy NP (732)	Atlantic Maritime	Atlantic
Kejimkujik NP (578)	Atlantic Maritime	Atlantic
Prince Edward Island NP (648)	Atlantic Maritime	Atlantic
La Mauricie NP (461)	Boreal Shield	Boreal
Mingan Archipelago NP (441)	Boreal Shield	Boreal
Pukaskwa NP (545)	Boreal Shield	Boreal
Terra Nova NP (506)	Boreal Shield	Boreal
Banff NP (910)	Montane Cordillera	Pacific
Glacier NP (634)	Montane Cordillera	Pacific
Mount Revelstoke NP (435)	Montane Cordillera	Pacific
Pacific Rim NP (436)	Pacific Maritime	Pacific
Yoho NP (658)	Montane Cordillera	Pacific
Elk Island NP (482)	Boreal Plains	Prairies
Grasslands NP (427)	Prairies	Prairies
Prince Albert NP (643)	Boreal Plains	Prairies
Riding Mountain NP (714)	Prairies	Prairies
Waterton Lakes NP (976)	Prairies	Prairies
1000 Islands NP (1631)	Mixed wood Plains	Woodland
Bruce Peninsula NP (877)	Mixed wood Plains	Woodland
Koffler Scientific Reserve (621)	Mixed wood Plains	Woodland
Point Pelee NP (858)	Mixed wood Plains	Woodland

**Fig 1:**
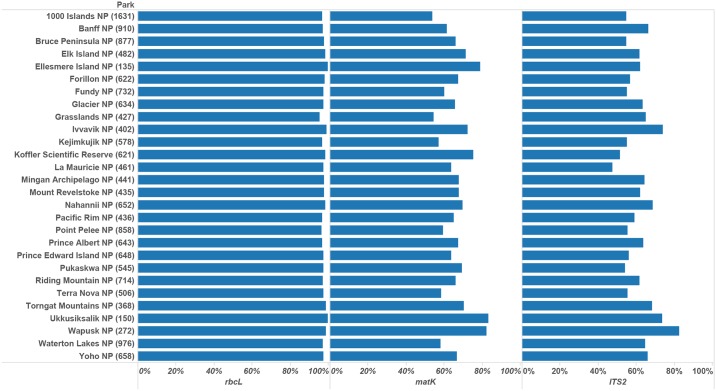
Coverage by barcode locus for the plant communities at 28 Canadian localities. The number of plant species present at each site is indicated in parentheses.

To test the taxonomic resolution of the DNA library we created ‘synthetic’ floras based on the checklist of vascular plants for each of 27 Canadian National Parks and the Koffler Scientific Reserve (KSR). Initial checklists were generated using the Parks Canada Biotics Web explorer at http://www.pc.gc.ca/apps/bos/bosfieldselection_e.asp, with more recent updates for Ellesmere, Ivvavik, Nahannii, Point Pelee, Torngat, Ukkusiksalik, and Wapusk National Parks (Bruce Bennett and Sergei Ponomarenko, personal communication). The species list for KSR was obtained from http://ksr.utoronto.ca/research/species-list/ksr-plant-list/. Plant species on the checklists were best represented by *rbcL* (> 95% coverage), followed by ITS2 and *matK* with comparable coverage (54–83% depending on the community; see Fig 1 for details). For the purpose of further analyses, the 28 checklists were clustered into six biogeographic regions: Arctic, Atlantic, Boreal, Pacific, Prairies, and Woodland (Table 1) representing 12 of the 15 terrestrial Canadian ecozones [32]. To ensure standardization of naming, all specimens and checklists used in this study followed the nomenclature accepted by VASCAN [31].

### Sequencing and analysis of libraries

#### Data validation

To reduce redundancy, identical sequences were clustered in UCLUST [33] and each cluster was parsed to its respective species (one species could be represented by more than one cluster). Sequences were then aligned using transAlign [34] for *rbcL* and *matK* (universal codon table), and MAFFT ver 7.221 for ITS2 under default parameters (FFT-NS-2 strategy) [35]. Maximum likelihood phylogenies were inferred for each alignment using RAxML Black box [36] on XCEDE via the CIPRES portal [37]. A dataset of 1074 species with records for all three gene regions was used to evaluate variation in taxonomic resolution (via BLAST and mothur) and phylogenetic metrics (MPD and MNTD). To estimate the number of unique sequences as a proxy for sequence variation, we clustered each marker at 100% using UCLUST [33].

#### Phylogenetic matrices

We calculated two metrics for each barcode region, mean phylogenetic distance (MPD) and mean nearest taxon distance (MNTD) [38] to examine their potential as predictors of the capacity of each region to resolve species. MPD is the average of the branch lengths (or distances) across all pairs of taxa in a phylogeny. It summarizes the overall phylogenetic diversity of a community and is influenced by the number of taxa in a tree [39]. By comparison, MNTD is an average of the distance between nearest neighbours so it describes the terminal phylogenetic structure. MNTD is the more appropriate measure of species resolution because it excludes internal nodes and instead calculates the mean distance between closely related species. Because both measures are influenced by polytomies in a phylogeny [40], we only included one representative per species to avoid bias created by an unequal number of sequences per species.

MPD and MNTD were estimated using the picante package [41] in R ver 3.2.0 [42]. The phylogenetic matrices for each barcode were calculated using the maximum likelihood tree, and were partitioned by family and genus. Regression analysis was used to determine if there was a correlation between each phylogenetic diversity metric and the number of sequences for a family. We also compared MNTD values for the three markers using a common set of genera to determine the strength of the correlation in their divergence values. To determine if significant differences existed between markers, Kruskal-Wallis (KW) tests followed by a Dunn’s posthoc were carried out in R ver 3.2.0 [42].

A similar analysis was conducted for each park community using RAxML-based trees to calculate MPD and MNTD partitioned by family. The percentage of congeners in the six large families with low MNTD (Asteraceae, Brassicaceae, Cyperaceae, Poaceae, Rosaceae, and Salicaceae) was evaluated for the datasets representing the three barcodes for the six biogeographic regions.

#### Taxonomic resolution

Our custom sequence library for Canadian plants was parsed based on the species present at each locality and the taxonomic resolution provided by each barcode was then evaluated using BLAST searches and by mothur in Qiime [43]. For both methods, the species known for each park were compared with the parent library to ascertain if barcode records allowed their identification to a family, genus, or species level. The resolution for species with multiple sequences was recorded as that where the taxonomic assignment for all individuals was consistent (e.g. if there were four sequences for species A and three were unambiguously identified to a species and one was to a genus, the recorded level of resolution would be to a genus). This approach generates a ‘worst case’ outcome for the capacity to identify a particular species. Mothur employs a distance matrix to assign a sequence (or cluster) to a species based on a parent library. For its use, identifications were predicted using a posterior probability cut-off of 0.95. We also report the true level of success of mothur by comparing the taxonomic identification assigned to a given sequence by mothur with its correct assignment. The data for each park was then used to generate a mean level of taxonomic resolution for each family, genus, and species. Data was checked for normality prior to conducting a Kruskal-Wallis (KW) test or one-way ANOVA to test for significant differences in species resolution among the three markers. Any significant test was followed up with the appropriate posthoc tests (Tukey’s HSD for ANOVA or Dunn for KW). The parks were then subdivided into six biogeographic regions (Arctic, Atlantic, Boreal, Pacific, Prairies, Woodland) and the data was pooled for each region to estimate the mean level of taxonomic resolution for the floras that were examined. After checking for normality, KW or one-way ANOVA was used to test for a significant difference in species resolution among the regions for a particular barcode marker. We also evaluated taxonomic resolution for the 1074 species with data for all three barcode genes to compare the mean of the parks and the performance of different markers using an identical set of taxa. The performance of the barcodes for 25 families with the most species was then compared based on the BLAST results to identify groups where barcodes delivered low taxonomic resolution. All statistical tests were performed in R ver 3.2.0 [42] with Bonferroni error corrections for multiple tests (adjusted p = 0.005).

## Results

### Clustering and phylogenetic matrices

**Fig 2:**
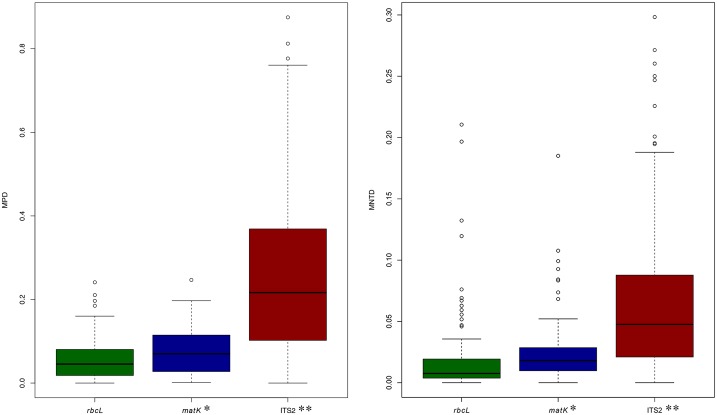
Boxplots of MPD and MNTD for *rbcL*, *matK*, and ITS2. Boxplots comparing MPD and MNTD for the vascular plant families of Canada for *rbcL*, *matK*, and ITS2. Significance (p–adjusted < 0.005) is indicated with an asterisk(s).

After the removal of identical sequences within any one species, the read library was reduced to 5919 sequences for *rbcL*, 2891 sequences for *matK*, and 4423 sequences for ITS2. The plastid markers were much less variable than ITS2 as evidenced when the read libraries were clustered at 100% identity which collapsed the sequence count when different species shared a particular sequence. This analysis showed that *rbcL* had considerably less sequence variation (2895 clusters; 5919 sequences) than *matK* (2145 clusters; 2891 sequences) while ITS2 was most diverse (4418 clusters; 4423 sequences). This pattern was reinforced by the global estimates for MPD and MNTD that rated ITS2 as the most variable marker followed by *matK* and *rbcL* (Fig 2, Table A and Table B in S1 File). For each measure, markers were significantly different from one another (KW and Dunn’s posthoc p < 0.0005).

The Asteraceae had low values for both metrics across all three barcodes (Table 2), but those for the family Salicaeae were exceptionally so for MPD (*rbcL* = 0.017; *matK* = 0.009; ITS2 = 0.036) and MNTD (*rbcL* = 0.0005; *matK* = 0.0007; ITS2 = 0.013). The latter result reflected the low MNTD values within *Salix* (41–90 species per region; *matK* and *rbcL* = 0.0005; ITS2 = 0.009; Table B in S1 File). The Asteraceae also had low MNTD (*rbcL* = 0.002; *matK* = 0.006; ITS2 = 0.021), strongly influenced by four genera with MNTD < 2 e–06. Interestingly, MPD did not predict low species resolution for Asteraceae (*rbcL* = 0.073; *matK* = 0.07; ITS2 = 0.373), because some long internal branches raised this measure (Table B in S1 File).

**Table 2:** The mean MPD and MNTD for 25 species-rich families with the number of sampled species.

Family	*rbcL*			*matK*			ITS2		
	No. of species	MPD	MNTD	No. of species	MPD	MNTD	No. of species	MPD	MNTD
**Amaranthaceae**	85	0.0889	0.0060	36	0.1395	0.0227	75	0.2660	0.0201
**Apiaceae**	98	0.0315	0.0037	59	0.0621	0.0106	66	0.3154	0.0469
**Asteraceae**	569	0.0729	0.0022	239	0.0699	0.0059	481	0.3732	0.0209
**Boraginaceae**	74	0.0579	0.0047	21	0.1065	0.0115	42	0.5789	0.0538
**Brassicaceae**	233	0.0320	0.0025	82	0.0700	0.0153	208	0.1887	0.0184
**Caprifoliaceae**	48	0.0756	0.0030	23	0.1138	0.0118	7	0.8123	0.3753
**Caryophyllaceae**	134	0.0805	0.0075	41	0.1841	0.0287	121	0.3109	0.0325
**Cyperaceae**	397	0.0329	0.0040	119	0.0552	0.0063	112	0.2724	0.0574
**Ericaceae**	71	0.0992	0.0120	54	0.1071	0.0135	61	0.1939	0.0236
**Fabaceae**	211	0.1602	0.0092	104	0.1291	0.0114	156	0.3646	0.0377
**Juncaceae**	69	0.0952	0.0040	39	0.1742	0.0092	19	0.2826	0.0081
**Lamiaceae**	92	0.0616	0.0036	22	0.0844	0.0203	36	0.5781	0.1954
**Onagraceae**	59	0.0558	0.0036	22	0.0543	0.0150	54	0.1015	0.0078
**Orchidaceae**	61	0.0690	0.0125	24	0.1148	0.0234	50	0.6388	0.0612
**Orobanchaceae**	79	0.0700	0.0077	24	0.0927	0.0274	49	0.3724	0.0516
**Plantaginaceae**	95	0.0942	0.0099	37	0.1654	0.0326	77	0.4472	0.0388
**Poaceae**	438	0.1185	0.0043	242	0.0963	0.0083	235	0.3941	0.0391
**Polemoniaceae**	43	0.0341	0.0044	7	0.0885	0.0288	39	0.1060	0.0165
**Polygonaceae**	85	0.0638	0.0053	25	0.1537	0.0521	41	0.5349	0.1005
**Primulaceae**	40	0.0906	0.0116	16	0.1063	0.0196	37	0.2703	0.0496
**Ranunculaceae**	120	0.0943	0.0057	39	0.1539	0.0181	93	0.3356	0.0341
**Rosaceae**	276	0.1021	0.0030	128	0.1183	0.0059	200	0.3677	0.0340
**Salicaceae**	100	0.0172	0.0005	82	0.0093	0.0007	46	0.0362	0.0128
**Saxifragaceae**	72	0.0635	0.0057	42	0.1318	0.0158	63	0.3226	0.0355
**Violaceae**	35	0.0127	0.0026	12	0.0310	0.0138	29	0.1021	0.0262
**Top 25 Mean**	143.36	0.0710	0.0056	61.56	0.1045	0.0171	95.88	0.3427	0.0559
**All Families Mean**	28.51	0.0552	0.0180	16.95	0.0770	0.0249	24.54	0.2823	0.0987

High phylogenetic diversity for families lacking genera with a low MNTD or MPD is a strong predictor of strong species resolution. For example, the Caryophyllaceae and Fabaceae have high MPD and MNTD for *rbcL* (MPD = 0.081, 0.160; MNTD = 0.008, 0.009 respectively; Table 2), but several of their genera have near zero values for both metrics (< 0.001; Table B in S1 File) suggesting that these lineages will have much lower species resolution than highly variable genera. By contrast, nearly all genera of the Orchidaceae and Primulaceae have high MPD and MNTD, ensuring high species resolution (see Table B in S1 File). Species resolution is also strong for the Lamiaceae (ITS2), Onagraceae (*matK*), and Polygonaceae (ITS2 and *matK*) (Table 2) due to their high genetic diversity. There was no correlation between the number of species in a family or genus and either MPD or MNTD (r^2^ < 0.05 for all comparisons). There was also no correlation between markers for MNTD values (r^2^ < 0.007 for all comparisons; S1 Fig).

MPD and MNTD were used to predict those parks and biogeographic regions where DNA barcodes would deliver poor taxonomic resolution. Both values were generally lower in the Arctic than in the other biogeographic regions for all three markers (see Table C in S1 File for details), suggesting that species resolution should be most challenging in the north (Table C in S1 File). These estimates of genetic diversity further predict that ITS2 will deliver the best taxonomic resolution followed by *matK* and *rbcL*.

### Taxonomic resolution

#### Overall

**Fig 3:**
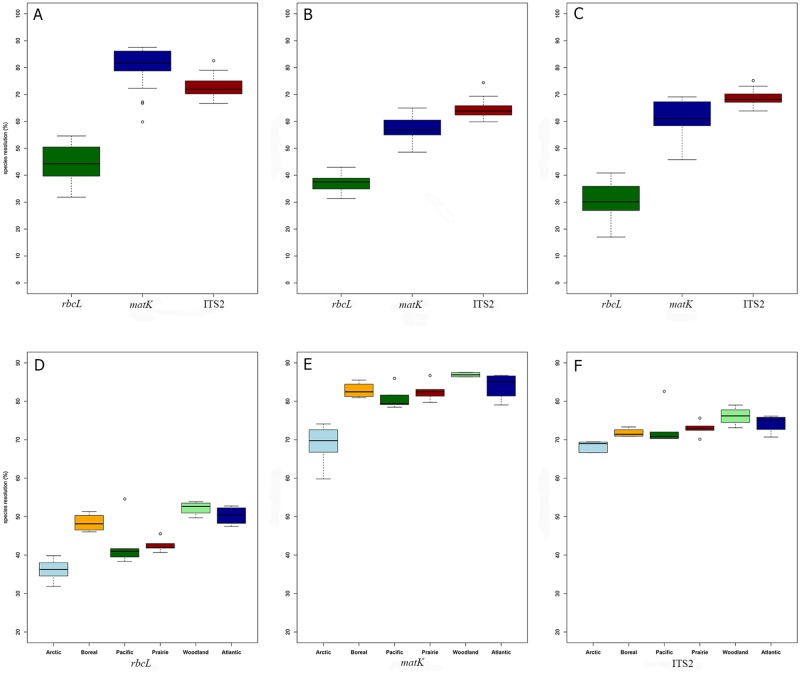
Species resolution for the three DNA barcodes (*rbcL*, *matK*, and ITS2). Species resolution for the three DNA barcodes (*rbcL*, *matK*, and ITS2) based on A) BLAST, B) mothur with a posterior probability cut–off 0.95 or C) the actual species resolution of mothur. Species resolution in the six biogeographic regions obtained with D) *rbcL*, E) *matK*, F) ITS2.

Performance comparison of BLAST and mothur in identifying plants from the 28 localities (Table 3; Fig 1) indicated that BLAST delivered higher species resolution for all three barcodes (Fig 3a–3c). When employing a posterior probability cut–off of 0.95, mothur underestimated the capacity to make species-level identifications, but overestimated it at a genus level Table D in S1 File). Both BLAST and mothur indicated that *rbcL* has the lowest species (45% and 31% with BLAST and mothur respectively) and generic (91% and 84% with BLAST and mothur respectively) resolution (Table D in S1 File), but diverged on which marker provides the highest species resolution. BLAST generates the highest species resolution with *matK* (80%) followed by ITS2 (73%). By comparison, mothur ranks ITS2 as the best barcode when resolving taxa with both posterior probability (ITS2 mean = 64% vs. *matK* mean = 58%) and true species resolution (69% versus 62%). Generic resolution was high for both *matK* (~96–98%) and ITS2 (96–99%) using either approach (Table 3). The difference in species resolution was significant between markers for both algorithms (p < 0.005; Fig 3a–3c). Analysis of the dataset consisting of 1074 species represented by all three barcodes generated similar results to the park data (Table D in S1 File). Since BLAST yielded the highest species resolution for each marker, these results were employed for the further analyses.

**Table 3:** Level of species resolution (%) for each barcode for BLAST and mothur. For mothur, species resolution is reported for both a posterior probability cut-off (0.95) and the true level of resolution.

Region	Park	Blast			Mothur PP			Mothur Actual		
		*rbcL*	*matK*	*ITS2*	*rbcL*	*matK*	*ITS2*	*rbcL*	*matK*	*ITS2*
Arctic	Ellesmere Island NP (135, 107, 84)	31.85	59.81	**66.67**	42.96	48.60	**64.29**	17.04	45.79	**67.86**
Arctic	Ivvavik NP (399, 292, 298)	34.59	66.78	**69.46**	36.09	55.14	**63.09**	24.81	51.71	**68.46**
Arctic	Nahannii NP (643, 456, 450)	36.86	**72.59**	69.11	36.39	54.82	**60.22**	25.51	55.92	**65.56**
Arctic	Torngat Mountains NP (364, 260, 252)	39.84	**72.31**	66.67	38.19	53.46	**61.11**	25.27	55.38	**63.89**
Arctic	Ukkusiksalik NP (149, 125, 111)	38.00	67.20	**69.37**	36.00	56.00	**69.37**	24.00	53.60	**71.17**
Arctic	Wapusk NP (269, 224, 225)	35.69	**74.11**	68.89	37.55	57.59	**62.67**	27.14	58.48	**70.22**
Atlantic	Forillon NP (611, 420, 355)	47.46	**79.05**	70.70	37.97	58.81	**62.82**	32.90	64.76	**67.04**
Atlantic	Fundy NP (714, 442, 406)	49.02	**83.71**	74.63	38.80	59.05	**66.50**	35.15	65.16	**67.98**
Atlantic	Kejimkujik NP (561, 332, 319)	52.76	**86.75**	75.55	41.35	62.35	**64.58**	40.64	**68.98**	65.83
Atlantic	Prince Edward Island NP (633, 414, 365)	51.82	**86.47**	76.16	39.97	61.84	**63.84**	39.18	**67.87**	67.40
Boreal	La Mauricie NP (450, 295, 220)	51.33	**83.39**	70.91	42.00	56.95	**65.91**	38.00	63.39	**68.18**
Boreal	Mingan Archipelago NP (432, 299, 285)	46.99	**80.94**	73.33	37.50	61.87	**65.96**	31.94	65.55	**70.88**
Boreal	Pukaskwa NP (532, 379, 296)	46.05	**81.53**	70.95	37.59	56.46	**62.50**	31.58	59.89	**68.58**
Boreal	Terra Nova NP (495, 297, 281)	49.29	**85.52**	71.89	37.98	64.98	**66.19**	33.94	68.01	**68.33**
Pacific	Banff NP (886, 561, 606)	38.37	**79.14**	70.30	33.97	55.44	**60.23**	25.96	58.29	**65.18**
Pacific	Glacier NP (618, 418, 404)	41.68	**78.47**	70.30	35.54	54.78	**63.86**	26.82	61.72	**66.58**
Pacific	Mount Revelstoke NP (427, 296, 271)	40.98	**79.39**	70.85	35.83	55.41	**63.47**	29.98	62.50	**67.16**
Pacific	Pacific Rim NP (423, 285, 258)	54.61	**85.96**	82.56	35.93	60.35	**74.42**	35.46	69.12	**75.19**
Pacific	Yoho NP (643, 440, 436)	39.50	**81.63**	72.02	33.59	56.92	**64.91**	27.84	59.86	**66.74**
Prairies	Elk Island NP (476, 345, 299)	41.81	**79.71**	73.58	32.77	53.62	**59.87**	29.41	57.68	**69.57**
Prairies	Grasslands NP (408, 233, 278)	41.91	**86.70**	70.14	31.37	52.79	**62.23**	29.66	58.80	**67.27**
Prairies	Prince Albert NP (623, 434, 410)	43.02	**82.49**	72.44	33.07	55.76	**61.22**	30.02	60.14	**70.24**
Prairies	Riding Mountain NP (696, 473, 443)	45.55	**83.09**	75.62	33.62	57.72	**62.75**	30.32	60.47	**69.53**
Prairies	Waterton Lakes NP (949, 568, 632)	40.67	**81.34**	72.94	34.25	54.23	**61.55**	26.98	59.86	**67.41**
Woodland	1000 Islands NP (1583, 880, 896)	52.18	**87.39**	79.02	38.66	60.68	**65.63**	36.39	67.05	**73.10**
Woodland	Bruce Peninsula NP (859, 580, 481)	49.71	**86.38**	75.88	39.12	60.17	**66.11**	36.55	67.76	**70.69**
Woodland	Koffler Scientific Reserve (612, 469, 320)	53.92	**86.35**	73.13	39.05	62.26	**64.69**	40.85	67.59	**68.13**
Woodland	Point Pelee NP (832, 513, 477)	53.13	**87.52**	76.52	39.90	63.16	**65.83**	37.38	69.01	**71.07**
**Parks mean**		44.59	80.20	72.48	37.04	57.54	64.14	31.10	61.58	68.54

#### Species resolution by family

**Fig 4:**
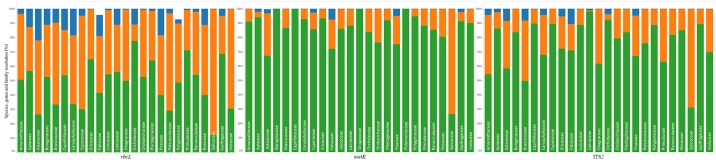
Level of taxonomic resolution provided by *rbcL*, *matK* or ITS2 for 25 families. Level of taxonomic resolution provided by *rbcL*, *matK* or ITS2 for 25 families of vascular plant that are species-rich in Canada. The three colours show the proportion of species identified to a family (blue), genus (orange) or species (green) level.

For most families, *matK* delivered the highest resolution followed by ITS2, but the two gene regions were complementary, jointly delivering 85% species resolution if two families were excluded (Salicaceae, Asteraceae) (Table 4; Fig 4). In fact, *matK* delivered perfect resolution for four families (Onagraceae, Polemoniaceae, Boraginaceae, Caprifoliaceae), while ITS2 did well for Lamicaceae (98%) and Orchidaceae (92%). By comparison, *rbcL* had low species resolution (<60%) for all families except Orchidaceae (78%), Ericaceae (65%), Plantaginaceae (64%), Primulaceae (71%), and Saxifragaceae (69%). Generic resolution was high for *matK* (98%) and ITS2 (97%) but slightly lower for *rbcL* (91%). Families with compromised generic resolution included the Asteraceae (*rbcL* = 78%; *matK* = 97%; ITS2 = 92%), Fabaceae (*rbcL* = 81%; *matK* = 93%; ITS2 = 89%) and Poaceae (*rbcL* = 82%; *matK* = 95%; ITS2 = 95%) (Table 4; Fig 4).

**Table 4:** Percentage of taxonomic resolution by BLAST to family, genus and species. Taxonomic resolution for *rbcL*, *matK*, and ITS2 for 25 species-rich families.

Blast Family	*rbcL*			*matK*			ITS2		
	Family	Genus	species	Family	Genus	species	Family	Genus	species
Amaranthaceae	100.00%	96.48%	50.70%	100.00%	100.00%	91.09%	100.00%	95.65%	54.35%
Apiaceae	100.00%	87.34%	56.54%	100.00%	97.86%	94.12%	100.00%	97.62%	86.19%
Asteraceae	100.00%	77.97%	26.39%	99.76%	96.76%	67.17%	100.00%	91.50%	58.37%
Boraginaceae	100.00%	88.89%	52.59%	100.00%	100.00%	100.00%	100.00%	100.00%	83.56%
Brassicaceae	100.00%	90.29%	33.44%	100.00%	100.00%	86.60%	100.00%	91.81%	49.67%
Caprifoliaceae	100.00%	85.23%	53.69%	100.00%	100.00%	100.00%	100.00%	100.00%	89.66%
Caryophyllaceae	100.00%	81.49%	33.63%	100.00%	100.00%	92.83%	100.00%	95.61%	67.80%
Cyperaceae	99.94%	95.15%	30.08%	100.00%	97.29%	85.67%	100.00%	100.00%	89.19%
Ericaceae	100.00%	99.62%	64.82%	100.00%	100.00%	93.22%	100.00%	94.68%	72.22%
Fabaceae	95.66%	81.07%	41.42%	100.00%	92.48%	72.09%	100.00%	89.39%	70.88%
Juncaceae	100.00%	99.08%	54.29%	100.00%	100.00%	86.05%	100.00%	100.00%	88.81%
Lamiaceae	100.00%	88.93%	56.15%	100.00%	100.00%	88.04%	100.00%	99.04%	98.08%
Onagraceae	100.00%	99.55%	49.77%	100.00%	100.00%	100.00%	100.00%	100.00%	61.75%
Orchidaceae	100.00%	89.08%	77.59%	100.00%	100.00%	83.70%	100.00%	96.12%	92.24%
Orobanchaceae	100.00%	99.44%	52.54%	100.00%	100.00%	76.40%	100.00%	100.00%	79.35%
Plantaginaceae	100.00%	98.64%	64.07%	100.00%	100.00%	91.88%	100.00%	100.00%	83.54%
Poaceae	100.00%	81.48%	39.89%	100.00%	95.00%	75.25%	100.00%	95.23%	66.84%
Polemoniaceae	100.00%	96.77%	29.03%	100.00%	100.00%	100.00%	100.00%	100.00%	75.86%
Polygonaceae	92.61%	89.79%	48.59%	100.00%	100.00%	94.71%	100.00%	100.00%	88.67%
Primulaceae	100.00%	99.17%	71.07%	100.00%	100.00%	88.16%	100.00%	100.00%	63.11%
Ranunculaceae	100.00%	97.97%	54.07%	100.00%	100.00%	85.37%	100.00%	100.00%	81.75%
Rosaceae	100.00%	88.78%	39.86%	100.00%	100.00%	80.37%	100.00%	99.84%	85.02%
Salicaceae	100.00%	99.76%	12.24%	100.00%	100.00%	26.47%	100.00%	100.00%	30.96%
Saxifragaceae	100.00%	95.06%	68.72%	100.00%	97.69%	91.33%	100.00%	100.00%	89.24%
Violaceae	100.00%	100.00%	30.41%	100.00%	100.00%	90.00%	100.00%	100.00%	69.78%

Consistent with their low values for MNTD and MPD, species resolution was poor for the Salicaceae (<31%) and Asteraceae (<68%). Their low MPD and MNTD values also predicted that certain genes would fail to distinguish species of Violaceae (*rbcL*), Rosaceae (*matK*), and Onagraceae (ITS2). When accounting for genera with low MPD and MNTD within families, low species resolution was apparent for Fabaceae (*rbcL* and *matK*) and Caryophyllaceae (*rbcL*), while resolution was high for Orchidaceae (*rbcL*) and Primulaceae (*rbcL*). The lack of low resolution genera in the Polygonaceeae (*matK* and ITS2), Caprifoliaceae (ITS2 and *matK*), Polemoniaceae (*matK*), and Lamiaceae (ITS2) accounts for the relatively high success of barcoding in these taxa (Table B in S1 File; Fig 4).

#### Species resolution by region

When the 28 localities were organized into six biogeographic regions (Table 1), Arctic sites had significantly lower levels of species resolution than those in the other five regions (p < 0.05 for all markers; Fig 3d–3f) although results varied by marker. For *rbcL*, the Atlantic and Woodland regions have significantly higher taxonomic resolution than all others (p < 0.005) while Boreal and Pacific communities have significantly higher taxonomic resolution than Arctic and Prairie assemblages (p < 0.005). For *matK*, only Arctic communities have significantly lower species resolution (p < 0.005) than the other localities. Atlantic and Woodland have significantly higher resolution with ITS2 than the other regions (p < 0.005; Fig 3d–3f), as predicted by MPD and MNTD.

## Discussion

This study examined the effectiveness of DNA barcoding in the identification of plants from six biogeographic regions of Canada using both local alignment (BLAST) and phylogeny-based (MNTD and MPD) approaches. MPD and MNTD were first proposed as measures of phylogenetic diversity within a community [38], and have commonly been used to study community assembly [44; 45; 46]. MPD was previously used to compare substitution rates among plant families for three barcode regions (*rbcL*, *matK*, ITS2), and a positive correlation was reported between these rates and their capacity to discriminate species [26]. The present study extended this work by examining the utility of MPD and MNTD as predictors of species resolution for the same three gene regions.

Both MPD and MNTD indicated that ITS2 should deliver the best species resolution, an expected result given the higher rates of nucleotide substitution in nuclear than organellar genomes of plants [47; 48]. The prediction was supported when mothur was used to generate taxonomic assignments, but *matK* delivered the best species resolution with BLAST. Interestingly, BLAST yielded higher species resolution than mothur for all three markers, a result which was maintained even when analysis was restricted to the 1074 species with sequence data for all three regions. BLAST’s higher resolution is explained by its greater sensitivity to sequence length [49], as well its inclusion of indel variation, which phylogenetic approaches typically overlook. Although *matK* has less sequence variation than ITS2, it contains more indels which helped it to achieve higher species resolution with BLAST. Our results support the need for DNA barcoding to utilize phylogenetic methods that incorporate indels to maximize the resolving power of a given marker.

The genome compartment exposed to the highest intraspecific gene flow is generally the best suited for making species assignments because it reduces the likelihood that introgressed alleles will gain establishment and blur species diagnosis. Gene flow raises effective population size, reducing exposure to genetic drift, diminishing the chance of introgressed alleles gaining fixation in the gene pool, and increasing the probability that a particular gene will track species relationships [50]. Since the nuclear genome tends to experience greater dispersal and gene flow than the plastid genome, nuclear markers are generally more effective in species diagnosis than their plastid counterparts [50; 51]. Hence, the incorporation of a nuclear marker with the core (plastid) barcodes offers the advantage of compensating for situations where plastid markers fail to provide resolution. ITS2 did outperform its plastid counterparts in several species-rich families (i.e. Lamiaceae, Poaceae, Cyperaceae, and Saxifragaceae; Table 4) examined in this study. However, it was less effective in other families, likely reflecting incomplete lineage sorting stemming from its larger effective population sizes or, in rare cases, when plastid dispersal exceeds that of the nucleus [50; 51]. Despite these situations, the incorporation of ITS2 is preferable over additional plastid markers such as *psbA-trnH* because it occurs in all plant species which is not true for any plastid marker (including *rbcL* and *psbA-trnH*) and existing primers are nearly universal (as opposed to those for *matK*). Moreover, it delivers high resolution despite its short length (~350 bp), making it an ideal marker for studies using high-throughput sequencing platforms which cannot recover full-length sequences for longer barcodes such as *matK* (~800 bp) or those variable in length (*psbA-trnH*; 50–1000 bp).

The observed differences in taxonomic resolution for the three barcodes are undoubtedly influenced by selection, species demography, hybridization, lineage sorting, and phylogeographic structure (reviewed by [52]). The higher resolution of *matK* compared to *rbcL* reflects the different selective pressures acting on these genes. Because it encodes the large subunit of RuBisCo which has an essential role in photosynthesis, *rbcL* is under strong purifying selection in autotrophic plants [53] reducing its rate of evolution and constraining its utility for distinguishing closely related species. By contrast *matK*, an intron maturase involved in the splicing of group IIa introns, appears to be under relaxed purifying selection as evidenced by nearly equal substitution rates for all three coding positions [53; 54; 55; 56]. The relatively high rates of nucleotide substitution in *matK* compared to other plastid genes is useful for species delimitation, but a lack of conserved priming sites often undermines sequence recovery. Nuclear markers have a larger effective population size than plastid markers and tend to evolve more rapidly [47; 48]. The higher rates of nucleotide substitution and dispersal in plant nuclear genomes support their inclusion for plant DNA barcoding [51]. Additionally, the presence of multiple alleles for nuclear genes makes it possible to identify hybrids. Currently the only plant nuclear locus that meets barcoding criteria is ITS2 (see above) and its inclusion adds depth to barcode reference libraries by tracking a different genomic compartment.

The present analysis shows that MPD and MNTD are strong predictors of barcode resolution, identifying families and genera where taxonomic resolution is low. They were particularly useful in revealing genera with low resolution in families where divergences are high. For example, the Fabaceae has a relatively high MPD for both *rbcL* and *matK* but low species resolution, reflecting its inclusion of several genera (e.g. *Lupinus*, *Oxytropis*) with low MNTD. The latter genera explain the lower than average species resolution in this family for all markers, but this outcome was especially surprising for *Lupinus* because it was previously observed to have high genetic diversity in North America and low genetic diversity in the Andes due to a recent adaptive radiation [57]. Further research is needed to determine if a similar radiation occurred in North America. MNTD is a better predictor of species resolution than MPD because it quantifies the distance between pairs of closely related species and it is also less influenced by polytomies than MPD or PD [40]. As such, it is a better estimator of the efficacy of a given DNA barcode. The low correlation between MNTD values for the three barcode regions in different genera implies they are evolving independently (S1 Fig). As a consequence, the use of multiple barcode markers consistently improves taxonomic resolution because a particular marker can compensate for the deficits in resolution of its counterparts [7; 9; 10; 15; 17; 18; 19; 20; 21; 22; 23; 24; 25; 26]. This complementarity supports the use of specific barcodes that optimize species resolution for different groups [58; 59].

The patterns of variation in the phylogenetic matrices (MPD, MNTD) agree with the earlier conclusion [15; 16] that ITS2 has higher discriminatory power than *matK* or *rbcL* when specimens are analyzed against a local reference library. However, this conclusion may not extend to other situations, such as the present study, where taxonomic resolution is compared against a more comprehensive parent library, an approach which provides a ‘real world’ outcome of DNA barcoding. For example, Burgess et al. [23] reported 88% species resolution with *matK* and 80% with *rbcL* when identifications were driven by a barcode library comprised solely of plant species known from the site. Analysis of the same community with the barcode library for all Canadian plants lowered species resolution (i.e. 86% for *matK*, 54% for *rbcL*) but with the advantage that newly encountered plants would potentially be identified.

The low levels of sequence variation in several plant families likely reflects the joint impacts of polyploidization, hybridization, phylogeographic effects such as allele surfing during range expansions, and demographic effects including bottlenecks which reduce intra- and inter-specific variation [52; 60; 61]. These effects are more prominent in Arctic communities that might explain the frequent failure of both nuclear and plastid markers in discriminating species in this region [52; 62; 63; 64; 65]. Although these processes (singly or in combination) compromise the effectiveness of DNA barcoding in discriminating plant species, they do provide an opportunity to understand the factors that shape plant populations and genomes. While our dataset lacks the extensive sampling needed to differentiate between these processes, it does reveal taxa that require further study.

Among the 171 plant families in Canada, Salicaceae has the lowest species discrimination, largely due to the very limited genetic diversity among the 90 species of *Salix*. Its lack of variation in seven regions of the plastid genome was linked to frequent hybridization, incomplete lineage sorting, or repeated plastid capture events [66]. However, the same lack of resolution was observed with our ITS2 data and in a more detailed analysis of 22 species [26], indicating that the lack of divergence extends into the nuclear genome. This difficulty in differentiating *Salix* species using molecular markers may reflect hybridization, introgression, recent speciation, allele surfing via range expansion, and low rates of molecular evolution [52; 59; 67; 68; 69]. More extensive phylogenetic studies targeting nuclear markers or whole plastid genomes are necessary to clarify the processes driving the unusually low divergences in *Salix*.

The Asteraceae is another family where DNA barcodes deliver poor species resolution, but the underlying factors differ from those in the Salicaceae. The Asteraceae is a species-rich group that lacks reciprocal monophyly between some closely related species and genera [70], taxa that are difficult to differentiate with molecular data [71]. The Fabaceae also showed poor species resolution with all three loci (41–72%), an expected result given the number of poorly resolved genera in this family [72]. Although species resolution in certain groups may never be resolved by the targeted analysis of a few barcode loci, they do represent interesting models for testing the effectiveness of whole plastid genomes as a tool for species discrimination (reviewed by [58]).

When comparisons were extended across the six biogeographic regions of Canada, DNA barcoding delivered the poorest species discrimination in the Arctic, perhaps reflecting the higher incidence of congeners in Arctic communities (47–53%) than in other regions (39–48%; S2 Fig). As well, the arctic flora is rich in recently radiated congeners that have not achieved reciprocal monophyly [63; 65]. As a consequence, DNA barcoding delivered higher species resolution in the more floristically diverse regions. In fact, the most floristically diverse regions (Atlantic and Woodland) had the highest species resolution for all markers, suggesting that increased species diversity is correlated with genetic diversity. This difference likely reflects both demographic effects and shifts in community composition. For example, species of *Salix* comprise 8% of the flora near Churchill, reducing the overall success of barcoding at this arctic site [26]. We do not observe similar compositional biases in more floristically diverse communities that would influence overall barcoding success.

The present study has established that DNA barcoding delivers approximately 80% species resolution for plant communities in the temperate regions of Canada when either *matK* or ITS2 are employed, meaning that DNA barcoding can provide a standardized, rapid approach for ecological surveys in these settings. The same gene regions deliver near-perfect resolution to a generic level, a level of taxonomic placement useful for characterizing both past [73; 74] and present [11; 75] plant communities, for forensic applications [76; 77; 78], for validating the accuracy of specimen identifications in herbaria [28; 79], and for assessing herbivore diets [11; 80; 81; 82; 83]. The present study also demonstrates the ability of DNA barcoding to deliver particularly high levels of taxonomic resolution when comprehensive reference libraries are available for *matK* and ITS2, providing motivation for efforts to extend coverage for these genes.

## Conclusions

Comprehensive sampling (~ 96% taxonomic coverage) of the Canadian flora provided a unique opportunity to test the efficacy of DNA barcoding across a diverse set of communities. Analyses based on this library indicate that any one of the three barcode regions is very effective (>90%) in delivering a generic assignment while species resolution is often possible with ITS2 (72%) and *matK* (80%). BLAST demonstrated higher performance than mothur in assigning specimens to a species in all datasets, including those at a community level and for 1074 species with data for all three barcode regions. The higher performance of BLAST reflects its consideration of indel variation and absolute length of the marker, leading *matK* to deliver the highest resolution. Although ITS2 showed slightly lower performance, it has two important advantages; its short length makes it suitable for HTS-based applications, and it is readily recovered from diverse taxa, including vascular plants and fungi.

## Supporting Information

S1 Fig MNTD values for the three barcodes for genera. Comparison of MNTD values for the three barcode regions for genera of Canadian vascular plants. A) Three- dimensional scatter plot of 243 genera; B) Three-dimensional scatter plot of a subset of 171 genera with low MNTD values. The r^2^ is less than 0.007 for all comparisons.
(PDF)

S2 Fig The percentage of congeners for the six most species-rich families with low MNTD. The percentage of congeners for the six most species-rich families with low MNTD by barcode and region.
(PDF)

S1 File A supplemental file containing four tables. Raw MPD and MNTD values for the vascular plant families of Canada (Table A). Raw MPD and MNTD values for the vascular plant genera of Canada (Table B). The biogeographic region, number of families, MPD, and MNTD for the 28 Canadian localities employed as a basis to test barcode resolution (Table C). The mean taxonomic resolution to family, genus, and species for all 28 localities employed as a basis to test barcode resolution and for the subset of 1074 species with sequence data for all three barcode regions (Table D).
(XLSX)
