# Supplementary figures and images for: Testing the Efficacy of DNA Barcodes for Identifying the Vascular Plants of Canada
Source: PLoS One. 2017 Jan 10;12(1):e0169515. doi: 10.1371/journal.pone.0169515 (PMC5224991; doi:10.1371/journal.pone.0169515)

**A**

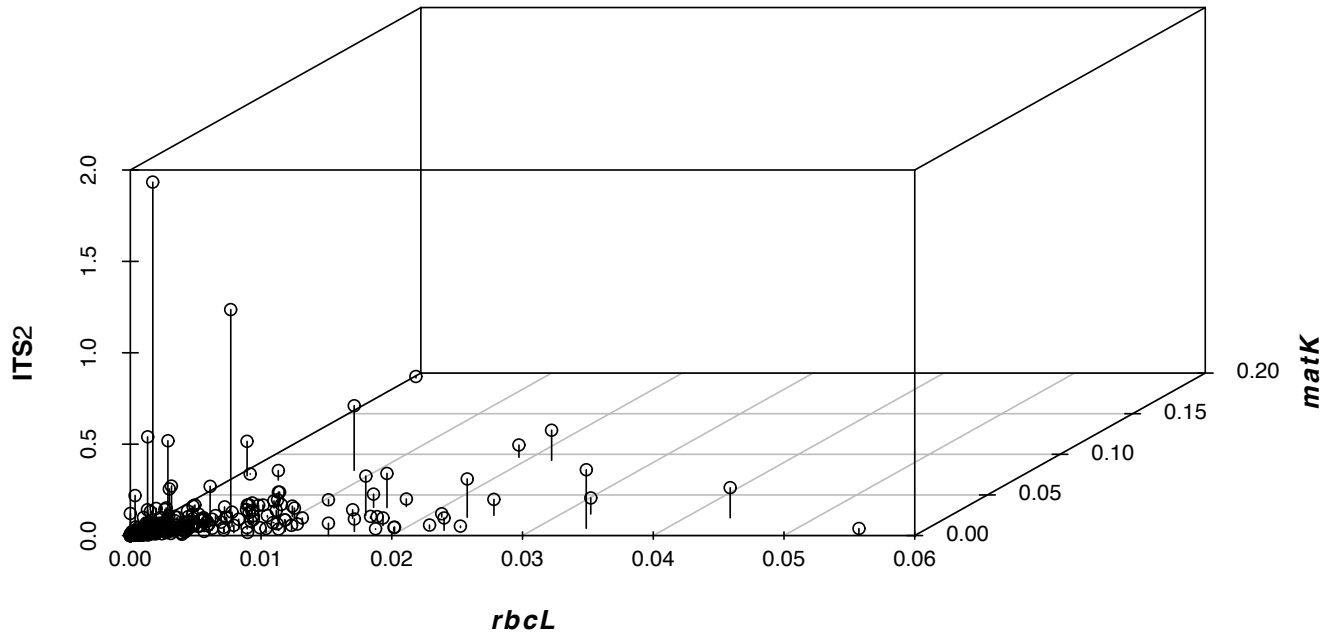

**B**

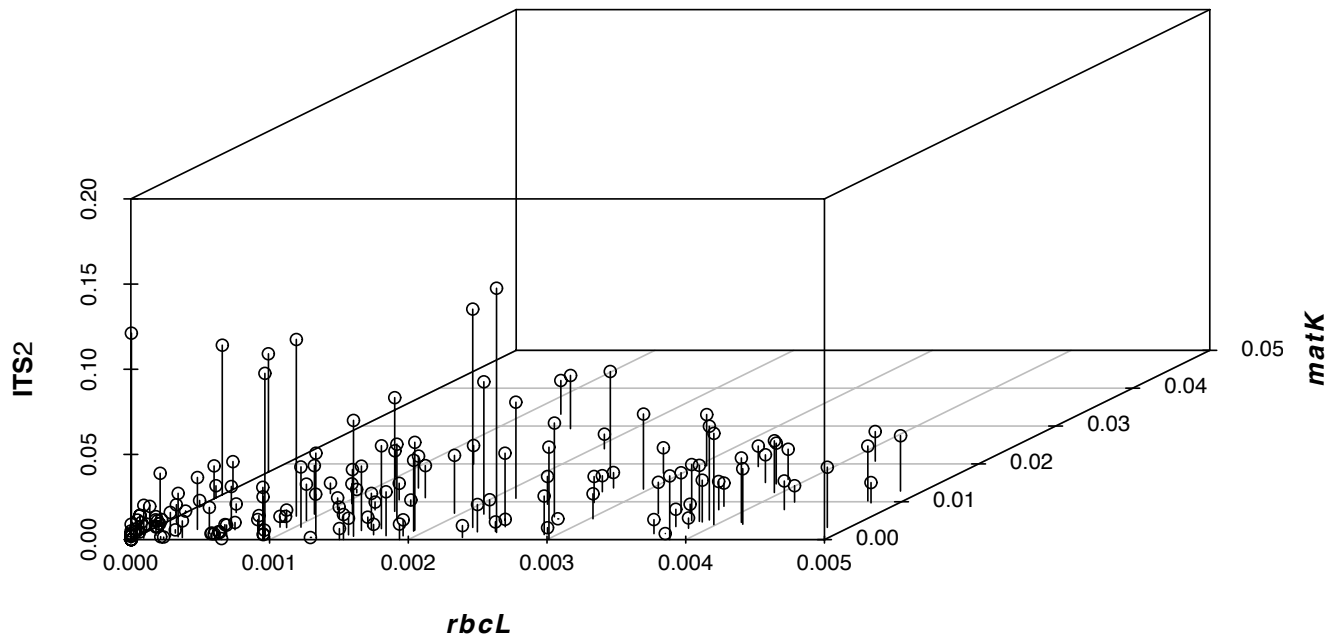

Supplement: S1 Fig — Comparison of MNTD values for the three barcode regions for genera of Canadian vascular plants. A) Three- dimensional scatter plot of 243 genera; B) Three-dimensional scatter plot of a subset of 171 genera with low MNTD values. The r2 is less than 0.007 for all comparisons. (PDF) [file pone.0169515.s001.pdf]

### ITS2 % of Congeners

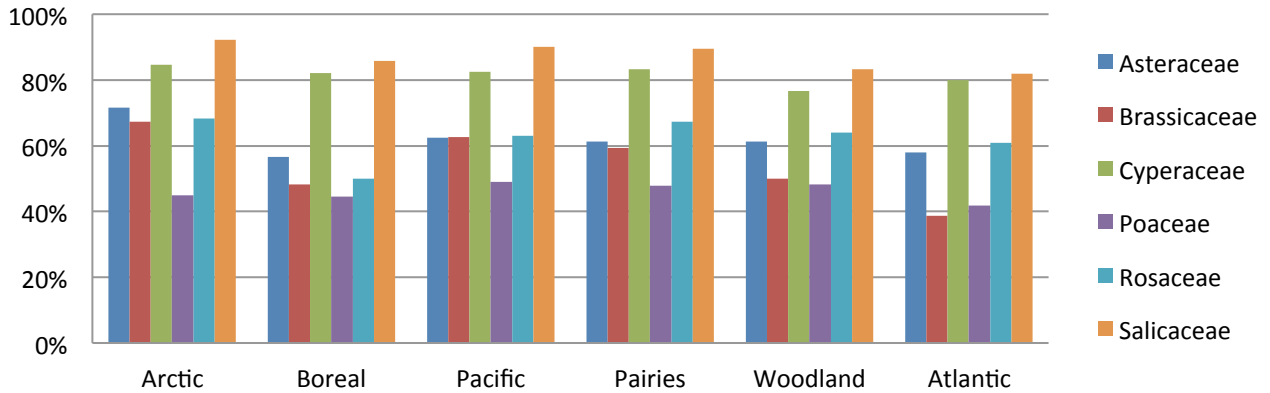

### *matK* % of Congeners

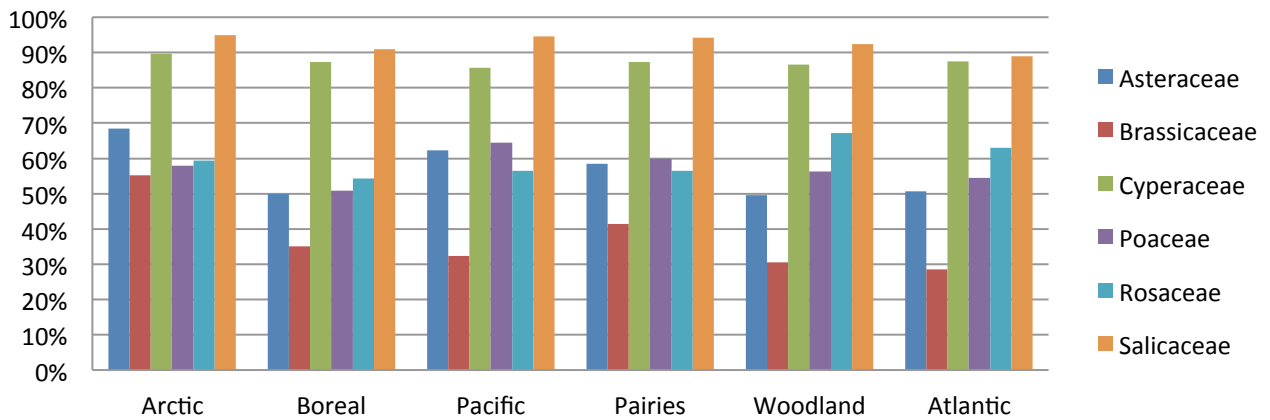

### *rbcl* % of Congeners

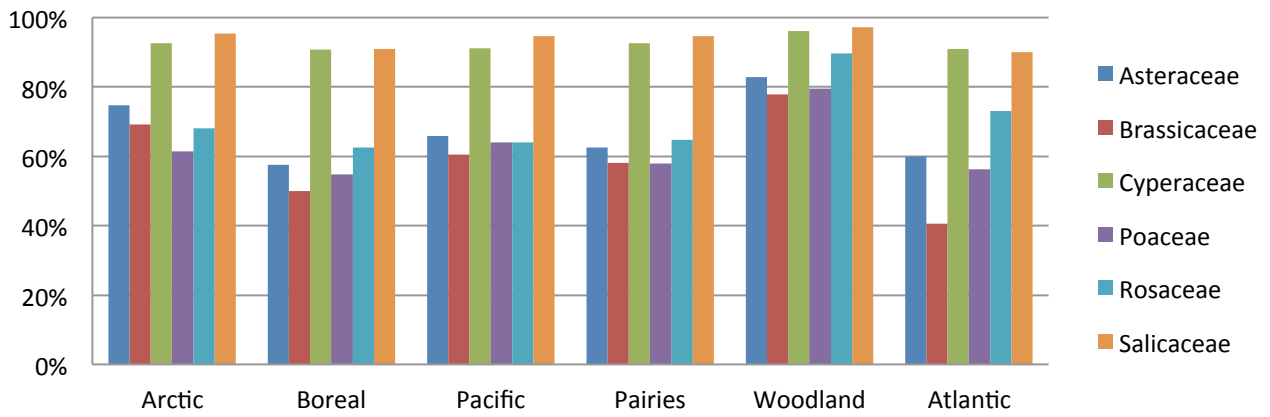

Supplement: S2 Fig — The percentage of congeners for the six most species-rich families with low MNTD by barcode and region. (PDF) [file pone.0169515.s002.pdf]
